# The Challenge of Understanding Gilmore’s Groin: An Analysis of Online Patient Resources

**DOI:** 10.7759/cureus.109872

**Published:** 2026-05-29

**Authors:** Victoria Byrne, Shane C Irwin, John P Gibbons

**Affiliations:** 1 Trauma and Orthopaedic Surgery, Mater Misericordiae University Hospital, Dublin, IRL; 2 Trauma and Orthopaedics, Mater Misericordiae University Hospital, Dublin, IRL

**Keywords:** accessible healthcare, athletic pubalgia, gilmore’s groin, online medical information, sports hernia, sportsman’s hernia

## Abstract

Objective

This study aimed to assess the quality and readability of online information available to patients on Google regarding Gilmore’s groin.

Methods

This descriptive cross-sectional study evaluated webpages identified through Google searches using the terms “sports hernia”, “athletic pubalgia”, “Gilmore’s groin”, “sportsman’s hernia”, and “hockey hernia”. The first page of results for each search term was screened. Duplicate links, non-functioning pages, and irrelevant results were excluded. Unique webpages meeting the eligibility criteria were analysed. Readability was assessed using the Gunning Fog Index (GFI), Flesch-Kincaid Grade Level (FKGL), and Flesch Reading Ease (FRE) score. Each webpage was further evaluated for source type, intended audience, presence of relevant media, inclusion of key clinical information, and quality using the Journal of the American Medical Association (JAMA) benchmark criteria. Descriptive statistics were used to summarise the findings.

Results

A total of 26 unique webpages were included. Hospital or clinic websites accounted for 13 (50%) webpages, and 16 (62%) were primarily directed toward patients. Relevant images were present in 11 (42%) webpages and relevant videos in three (11.5%). Information on cause and symptoms was provided in 26 (100%) webpages, investigations in 22 (85%), treatment in 25 (96%), and prognosis in 15 (58%). With respect to JAMA benchmarks, authorship was reported in 16 (61.5%) webpages, attribution in 13 (50%), disclosure in 21 (81%), and currency in 17 (65%). Mean readability scores were 11.5 for GFI, 9.9 for FKGL, and 43.5 for FRE, indicating that the material was generally written above the recommended reading level for patient education resources.

Conclusion

Online patient information on Gilmore’s groin is widely available but is typically written at a reading level that is too advanced for the general public. Improving readability while maintaining accuracy may enhance patient understanding, support shared decision-making, and improve access to health information.

## Introduction

Gilmore’s groin, also known as sports hernia or athletic pubalgia, is a condition characterised by chronic groin pain in athletes involved in activities requiring repeated twisting, turning, acceleration, and directional changes [1]. It is commonly associated with weakness or disruption of the posterior inguinal wall and adjacent soft-tissue structures [2,3]. Patients often report persistent groin pain that may radiate to the lower abdomen, adductor region, or scrotum in men [1,4]. The diagnosis of Gilmore's groin can be clinically challenging due to overlapping symptoms with other causes of groin pain, contributing to diagnostic ambiguity and variability in management approaches. Because symptoms may impair athletic performance, daily function, and quality of life, accurate diagnosis and effective patient education are important components of care [1]. Management is usually individualised and may include physiotherapy, activity modification, and in selected cases, surgical repair [5-7]. As diagnosis and treatment options can be complex, clinicians should ensure that patients understand the condition and are supported in shared decision-making [8].

Many patients seek health information online after receiving a diagnosis [9,10]. As a result, online information has become an increasingly influential component of health behaviour and understanding of treatment [11-15]. However, the value of online information depends not only on accuracy but also on readability. Materials written in overly technical language may be difficult for the average patient to understand, limiting informed participation in care [8,16,17]. While large language models (LLMs) and AI-based tools are increasingly used to summarise medical information, these systems typically rely on underlying web-based sources, making the quality and readability of primary online content critically important. 

The average reading ability in the United States is estimated to be around the seventh- to eighth-grade level, while patient education materials are generally recommended to be written at or below the sixth-grade level [18]. Previous studies have shown that online medical resources often exceed this threshold [16-18]. Gilmore’s groin can have a substantial effect on both a person’s physical health and emotional well-being, so strategies should be implemented to reduce the impact of this complex condition [19]. This study aimed to evaluate the quality and readability of online information accessible through Google searches on Gilmore’s groin. Google was selected as it remains the most commonly used search engine worldwide and reflects typical patient search behaviour when seeking health information. 

This article was featured in the best poster section at the 2024 EFORT Meeting (the 25th Annual Congress of the European Federation of National Associations of Orthopaedics and Traumatology) on May 22-24, 2024, in Hamburg, Germany. It was also shortlisted for the Jacques Duparc International Award. This article was presented at the XXXIII Waterford Surgical October Meeting on October 12, 2024, in Waterford, Ireland.

## Materials and methods

Study design

This was a descriptive cross-sectional study of publicly available online information on Gilmore’s groin.

Search Strategy

A Google search was performed using the following terms: “sports hernia,” “athletic pubalgia,” “Gilmore’s groin,” “sportsman’s hernia,” and “hockey hernia.” The search was conducted on 02 January 2025. Searches were performed using a standard web browser in a non-personalised setting, with no active user login, to minimise the influence of prior search history and personalised results. Only results appearing on the first page of Google for each search term were screened, in keeping with prior methodology used in studies evaluating online health information [18,20]. The first page was chosen because most users do not proceed beyond the initial search results. Although search engine results may vary based on location, time and algorithm updates, this approach was designed to replicate a typical patient search journey. 

Eligibility criteria

Webpages were included if they were accessible to the public without subscription or login, were written in English, contained information relevant to Gilmore’s groin or synonymous terms, and were retrievable from the first page of Google results for the selected search terms. Webpages were excluded if they were duplicate links, were broken or inaccessible, consisted only of video hosting pages without substantive text, were scientific journal articles aimed primarily at professionals rather than the general public, or were not directly relevant to the condition under investigation.

Study sample

**Figure 1:**
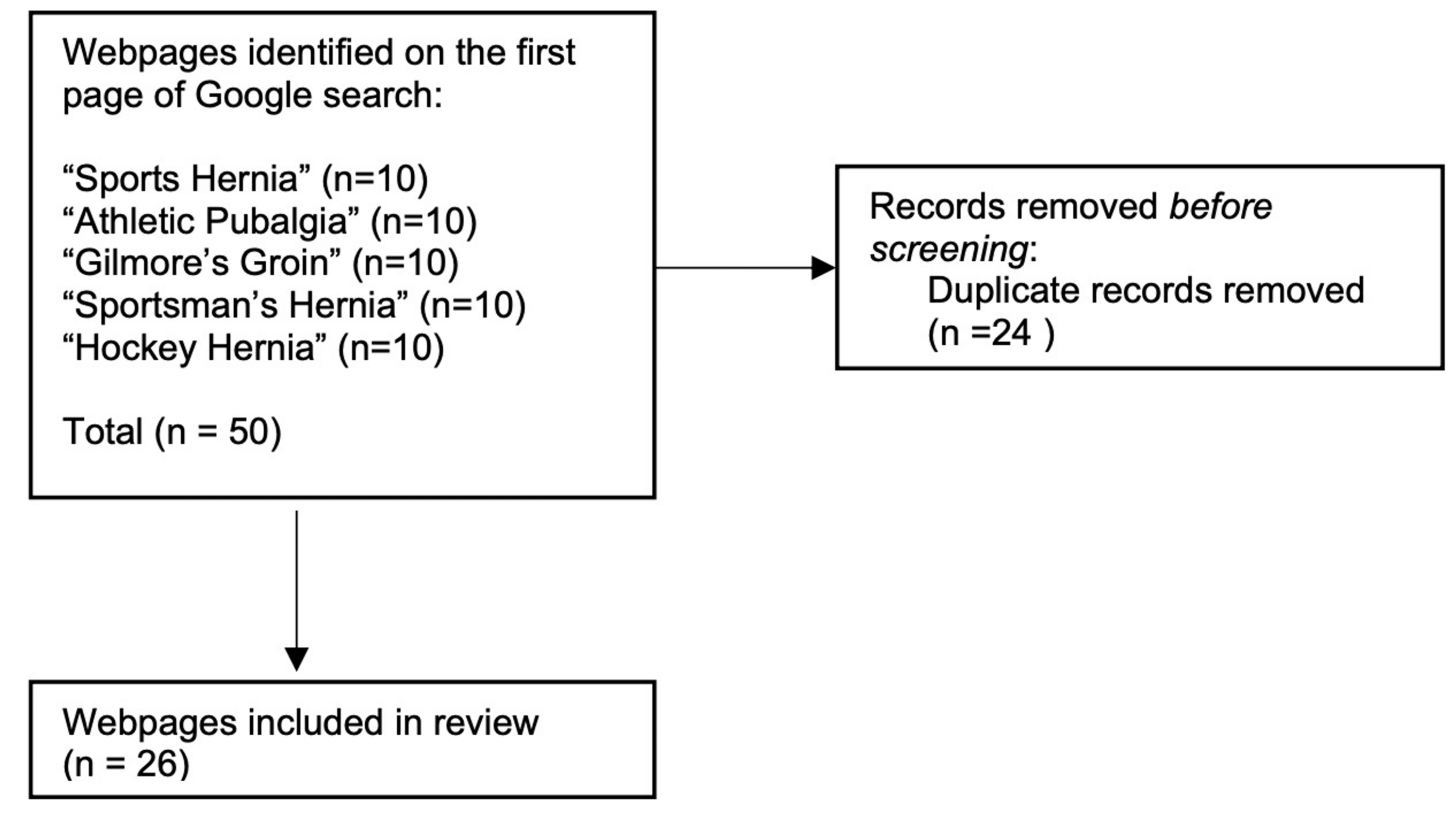
Flowchart of the search strategy n= number

The unit of analysis was the individual webpage/URL. After screening and duplicate removal, 26 unique URLs were included in the final analysis (Figure 1).

Sample size considerations

No formal sample size calculation was performed. The study sample was determined by the number of eligible unique webpages retrieved from the predefined search strategy. This approach is consistent with prior descriptive studies of online health information quality and readability [18,20].

Data collection parameters

For each included webpage, the following variables were recorded: source type (hospital/clinic website versus private/non-clinical source), intended audience (patient versus medical/professional), presence of relevant images, presence of relevant videos, whether the webpage included information on cause, whether it included symptomatology, whether it included investigations, whether it included treatment, and whether it included prognosis.

Readability assessment

Readability was assessed using the online Readable platform [21]. Three indices were recorded for each webpage: Gunning Fog Index (GFI), Flesch-Kincaid Grade Level (FKGL), and Flesch Reading Ease (FRE). Higher GFI and FKGL scores indicate more complex text, while lower FRE scores indicate more difficult reading material.

Quality assessment

Webpage quality was assessed using the Journal of the American Medical Association (JAMA) benchmark criteria, which evaluate four domains: authorship, attribution, disclosure and currency [22,23]. Each criterion was scored as present or absent, giving a total possible JAMA score ranging from 0 to 4.

Statistical analysis

Descriptive statistics were used. Categorical variables were summarised as frequencies and percentages, and continuous variables were summarised as means, standard deviations, and 95% confidence intervals. Statistical analysis was performed using Microsoft Excel (Microsoft Corporation, Redmond, WA, USA) and IBM SPSS Statistics for Windows, Version 29.0.1.1 (IBM Corp., Armonk, NY, USA).

Ethical considerations

This study analysed publicly available online content and did not involve human participants, patient data, or identifiable information. Formal ethical approval was therefore not required.

## Results

A total of 26 unique webpages were included in the final analysis after duplicate removal.

Webpage characteristics

Of the 26 included webpages, 13 (50%) were from hospital or clinic websites. In terms of intended readership, 16 (62%) were directed primarily toward patients, while the remainder were more professionally oriented. Relevant images were present in 11 (42%) webpages, and relevant videos were present in three (11.5%).

Clinical content

**Figure 2:**
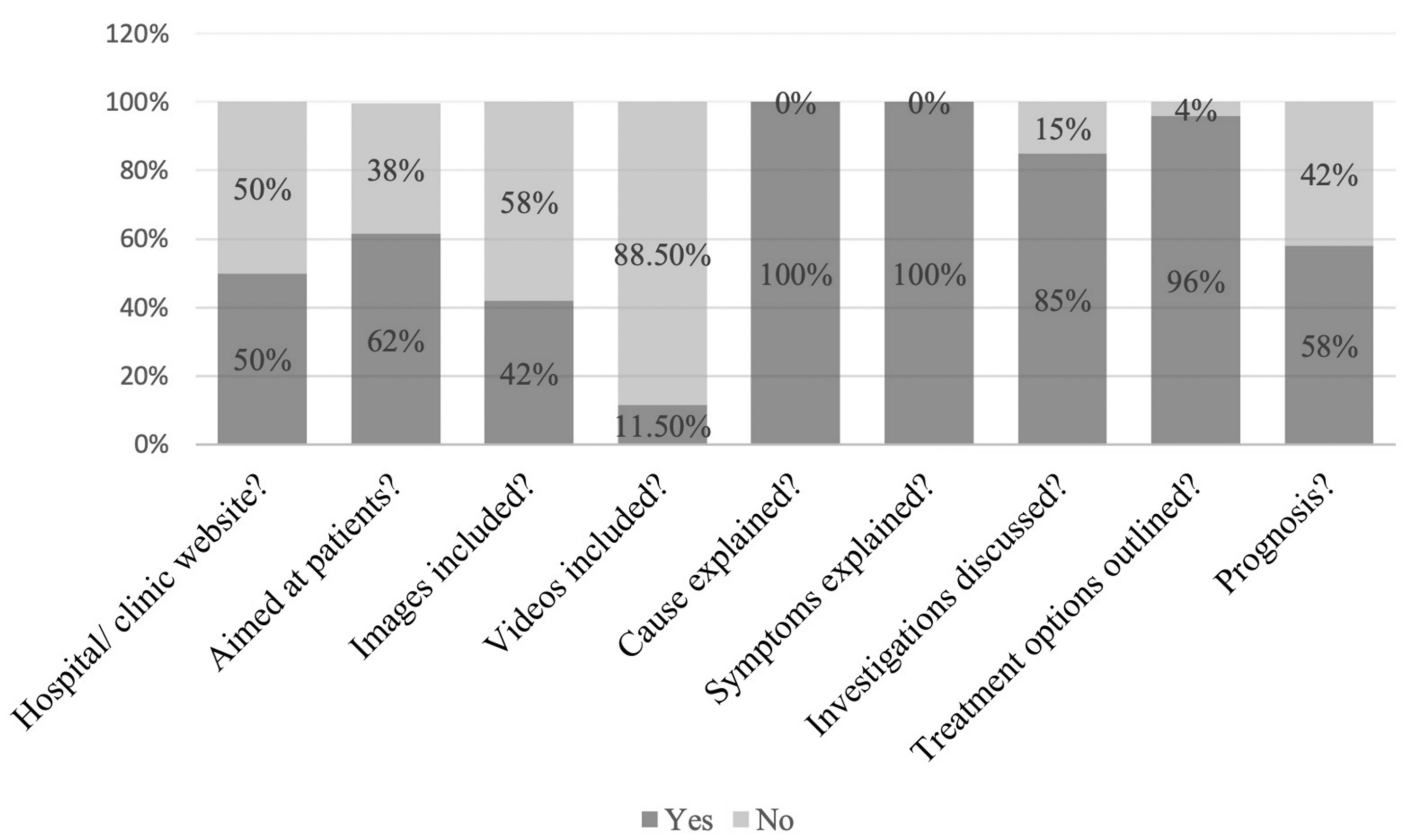
Results of the search quality

All included webpages, 26 (100%), provided at least some explanation of the cause of the condition and described associated symptoms. Information on investigations was present in 22 (85%) webpages, treatment options in 25 (96%), and prognosis in 15 (58%). These findings suggest that while most webpages addressed the core clinical features of Gilmore’s groin, prognosis was less consistently discussed (Figure 2).

JAMA benchmark criteria

**Figure 3:**
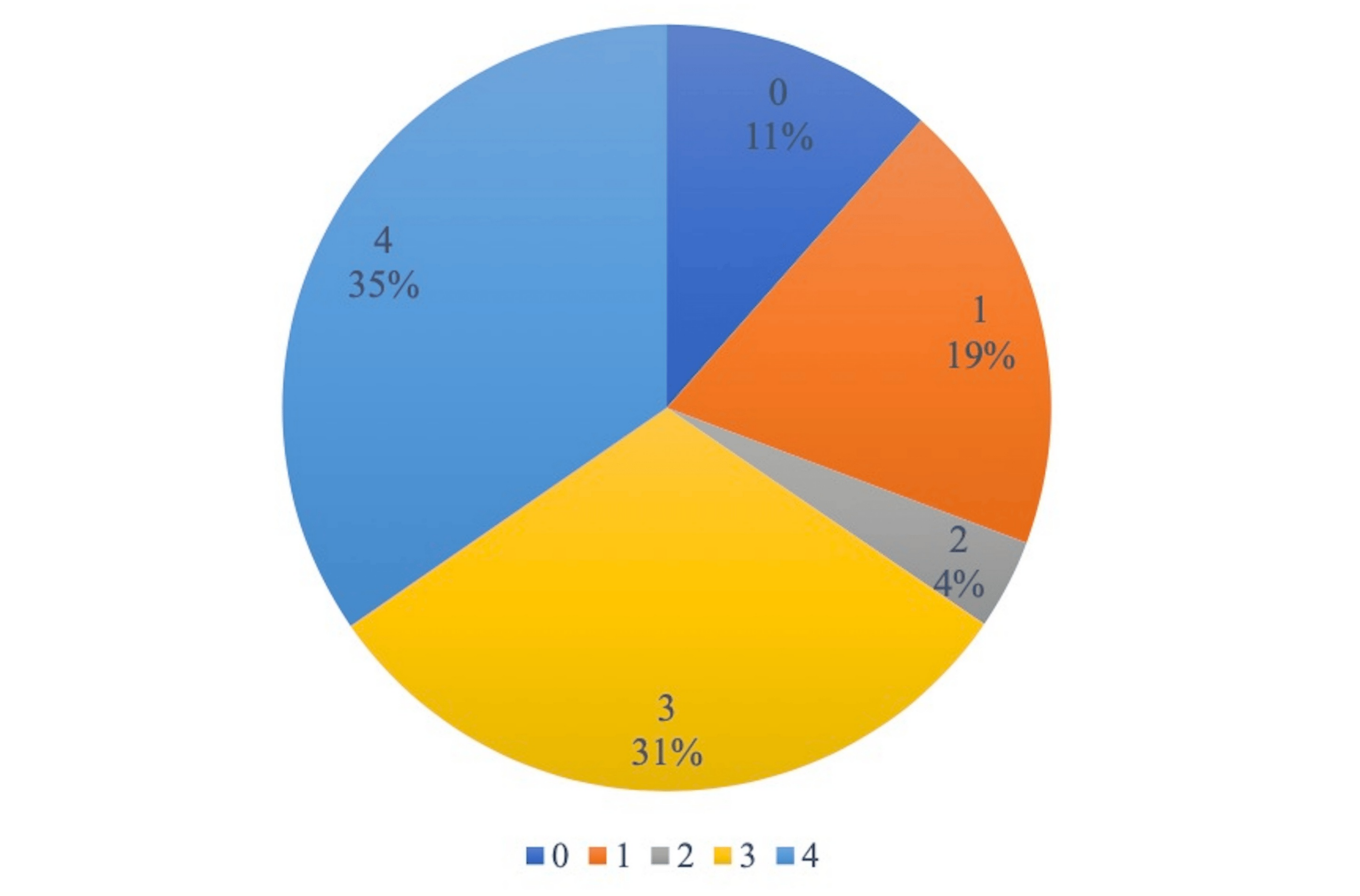
JAMA benchmark criteria assessment Percentage of webpages satisfying the Journal of the American Medical Association (JAMA) benchmark criteria (0-4)

With regard to quality indicators, authorship was identified in 16 (61.5%) webpages, attribution in 13 (50%), disclosure in 21 (81%), and currency in 17 (65%). When total JAMA benchmark scores were calculated, three (11.5%) webpages scored 0, five (19.2%) scored 1, one (3.8%) scored 2, eight (30.8%) scored 3, and nine (34.6%) scored 4. Thus, while a proportion of webpages met most of the JAMA quality domains, a notable number lacked key markers of transparency and reliability (Figure 3).

Readability

The mean GFI score was 11.5 ± 2.5 (95% CI: 10.5-12.6), indicating fairly difficult reading material. The mean FKGL score was 9.9 ± 1.5 (95% CI: 9.25-10.5), again suggesting a reading level above recommended thresholds for patient education. The mean FRE score was 43.5 ± 12.7 (95% CI: 38.4-48.7), corresponding to difficult text that would generally be more suitable for readers with college-level comprehension. Taken together, these findings indicate that most online information identified in this search would likely be challenging for the average patient to read and understand.

## Discussion

This study found that online information on Gilmore’s groin available through Google was often clinically relevant but generally difficult to read. The findings also need to be interpreted in the context of evolving digital health tools. AI-based summarisation platforms and large language models may improve accessibility by presenting simplified interpretations of complex medical information. However, these tools rely heavily on the quality of the source material, meaning that poor readability and transparency at the source level may still affect the accuracy and clarity of the generated summaries. Although most webpages addressed causes, symptoms, investigations, and treatment, readability scores consistently exceeded accepted recommendations for patient education material.

The observed readability burden is consistent with the broader literature on online health information. Prior studies in orthopaedics, sports medicine, radiology, and general health communication have repeatedly shown that web-based patient resources are frequently written above the recommended sixth-grade level [16-18,24,25,26]. In our study, the mean GFI of 11.5 and the mean FKGL of 9.9 suggest that the average webpage required reading skills at approximately a senior high school level or above. The mean FRE of 43.5 similarly indicates difficult material. This is substantially above the level generally advised for patient-facing information. Table 1, adapted from the table outlined by Ma Y et al., further demonstrates the readability score, equivalent readability level, and corresponding United States (US) school grade level [26].

**Table 1:** Readability score and equivalent readability level: US school grade level FRES: Flesch Reading Ease Score, FKGL, Flesch-Kincaid Grade Level, GFOG: Gunning Fog Index

Readability	Equivalent grade level in the US	FRES	FKGL	GFOG
Extremely easy	4th or below	Over 100	0	0-6
Very easy	5th	90-100	1-5	
Easy	6th	80-89		
Fairly easy	7th	70-79		
Standard	8th-9th	60-69	6-8	7-8
Fairly difficult	10th-12th	50-59	9-14	9-12
Difficult	College	30-49		13-16
Very confusing	Above college	0-29	≥15	17-19

These findings are important because patients regularly consult the Internet after receiving a diagnosis [9,10,27,28]. Online information can shape understanding of symptoms, expectations of treatment, and decision-making behaviour [15,27,28]. If the available resources are too complex, patients may misunderstand their condition or struggle to engage meaningfully in shared decision-making [29]. This is particularly relevant for Gilmore’s groin, where diagnosis can be nuanced and treatment may involve prolonged rehabilitation or surgery.

The quality analysis showed a mixed picture. While a substantial proportion of webpages performed reasonably well on JAMA criteria, not all sources disclosed authorship, attribution, or publication date. This is consistent with previous studies showing that online health resources may vary considerably in transparency and reliability [16,22-24,26]. In practical terms, a webpage may contain broadly correct information yet still provide limited reassurance regarding who wrote it, what evidence supports it, or whether it is up to date.

Comparison with earlier studies suggests that poor readability is not unique to this topic. Ahmed et al. found similar shortcomings in online concussion resources, while Irwin et al. reported that internet information related to ankle arthrodesis was also difficult for the general public to read [16,18]. Thomas et al. likewise demonstrated that online patient education materials for paediatric ACL tears often exceeded recommended reading levels [24]. Our findings extend this pattern to online information related to Gilmore’s groin.

From a clinical perspective, these results reinforce the need for clinicians to guide patients toward understandable and trustworthy resources. Practical strategies to improve patient education include reducing medical jargon, using shorter sentences, incorporating visual aids, and providing clear summaries. Healthcare providers may also consider actively recommending high- quality, accessible resources to patients.

Additionally, future research should explore how AI-generated summaries influence patient comprehension, decision-making and health literacy in comparison to traditional web-based resources.

Limitations

This study has several limitations. First, only the first-page Google results were analysed, which may not reflect the full range of available online information. The use of a single search engine may limit generalisability, as patients may also access information through alternative platforms, including social media and AI-based tools. However, this approach reflects typical user behaviour. Second, Google search results vary by geography, time, and personalisation, so the results may differ if the search is repeated. Although steps were taken to minimise personalisations, some variability in results is unavoidable. Third, readability formulas estimate textual difficulty but do not directly assess factual accuracy, user comprehension, or usability. Furthermore, this study did not evaluate AI-generated summaries or interactive digital tools, which are increasingly used by patients to access health information. Finally, the study included English-language webpages only and may not generalise to other languages or healthcare contexts.

## Conclusions

Online information about Gilmore’s groin on Google is often written at a level too advanced for the average reader. While emerging technologies, such as AI-based summarisation tools, may help improve accessibility, ensuring that primary online resources are clear, accurate, and readable remains essential. Although many webpages contain useful clinical information, readability remains a substantial barrier to patient understanding. Improving the clarity, transparency, and accessibility of these resources may help support patient education, shared decision-making, and engagement in treatment.
